# Use and users of the online self-management training “Living with food allergy”

**DOI:** 10.1186/2045-7022-5-S3-P154

**Published:** 2015-03-30

**Authors:** Harmieke Van Os-Medendorp, Anouska Michelsen-Huisman, Marieke Pronk-Kleinjan, André Knulst

**Affiliations:** 1 University Medical Centre Utrecht, Utrecht, The Netherlands

## Background

An online self-management training "Living with food allergy" has been developed for patients with a doctor's diagnosed food allergy (FA) to increase patient's self-management. A multidisciplinary team for food allergy developed the training. The online training consists of information, video's, patient's stories and exercises with feedback. The modules are “What is FA”; “How is it diagnosed”; “What to do in case of allergic reaction”; “Diet & food allergy”; “Cross-reactivity”; “Coping with FA in daily life”. The training was launched at the end of 2010, first for patients of the UMC Utrecht and later for all patients in the Netherlands. Since 2013 data about use of different modules are available.

## Methods

An explorative study was carried out under adult patients with a food allergy who used the online training. Use of the different modules of the online training and characteristics of users were measured. Relations between number of logins and patient characteristics, scores on food allergy related quality of life and scores on domains of self-management (as measured with the health education impact questionnaire) were studied.

## Results

A total of 214 adult patient were referred to the training, of them 74% was woman and 92% was referred by our own university hospital. Fifty-eight percent of all patients logged in at least one time. Data of use of the training of 30 patients were available for analyzes. In total they logged in 65 times. All modules were more or less equally used, except for the module cross reactivity which was less used. Most hits were for information-sections, exercises and video's. Forty-five patients filled in questionnaires; they had a mean of 3,5 (sd 1,9) food allergies and 33 (73%) were prescribed an adrenaline autoinjector. A significant moderate correlation (ρ: -0.36; p.02) was shown between number of logins and lower scores of the domain 'social integration and support'. No relation was found between the number of logins, age, sex, number of food allergies, adrenaline autoinjector and scores on food allergy related quality of life.

## Conclusion

The online self-management training "Living with food allergy" is mostly used by patients of a university centre; all modules seemed to be relevant, but there is room for improvement in use of the training. Lower social integration and support was related to higher use of the training. Further research is needed to the implementation of this online training in usual face-to-face care.

